# Supplementary material for: Patterns of Intron Gain and Loss in Fungi
Source: PLoS Biol. 2004 Nov 30;2(12):e422. doi: 10.1371/journal.pbio.0020422 (PMC532390; doi:10.1371/journal.pbio.0020422)
Supplement: Table S1 — Also available at http://genes.mit.edu/NielsenEtAl/. (4.3 MB ZIP). [file pbio.0020422.st001.zip › NielsenEtAl/html/1425.html]

AN0306.1.NCU03050.1.MG04745.1.FG07054.1


```
 CLUSTAL W (1.82) Multiple Sequence Alignments - Introns Inserted


Sequence 1: NCU03050.1	310 aa
Sequence 2: MG04745.1	319 aa
Sequence 3: FG07054.1	336 aa
Sequence 4: AN0306.1	320 aa
Alignment Length: 339 aa
Number Identitical Residues: 216 aa
Alignment Score (without introns) 9620


MG04745.1 	MLLLDYQNVLIQSVLTERFS-G2APPVSIDQTVSDFDGVIFHISTPESKTKILVSLQIRC
NCU03050.1	MLLLDYQNVLIQAVLTERFAPG2ASPVNIDQTVSDFDGVVFHISTPESKTKILVSLQIRC
FG07054.1 	MLLLDYQNVLIQSVLTERFS-G2APPASIDQTVSDFDGVTFHISTPETKTQVLLSIQIRC
AN0306.1  	MLLLDYENVLIRSLLTERFS-G2APPVSIDQIVSDFDGVTFHLSTPEVKTKILISLNVKC
          	******:****:::*****: * *.*..*** ******* **:**** **::*:*::::*

MG04745.1 	YKDLVKYGAEDVLNREYGQYVVPPEPGYDFSVMVDLENLPSEPE1AREELVKKISLLKRN
NCU03050.1	FQDLVRYGAEQVLNREYADLVVPPEAGYDFSIQVDLENLPAEQE~ARDALVMKIALLKRN
FG07054.1 	FPDLVKYGAEEVLQREYGDYVTAVEPGYDFSVLVDLENLPESKE1ERNELALKFALLKRN
AN0306.1  	FRELVQYGAQQVLEREYGPYIVAPEPGYDFSVLIDLENLPDDQT~AREDLITRLALLKRN
          	: :**:***::**:***.  :.. *.*****: :****** .    *: *  :::*****

MG04745.1 	AMAAPFEHAYAEHYKLKEEASKYTSEEAPQGIREGGEVMAIHYREEEAIYVKASFDRVTV
NCU03050.1	AMAAPFEQAYQEHYALKEEASKFTSEEAPQGVREGGEVKAIHYREEEAIYVKASHDRVTV
FG07054.1 	AMAAPFEQAYKEHYALKEEASKFTSEDAPQGIREGGEVKAIHYREEEAIYVKASHDRVTV
AN0306.1  	AMAAPFEKAFDEFAQLAEEASKYTSEAAPAGVAEGGEVMAIHYREEEAIYIKASHDRVTV
          	*******:*: *.  * *****:*** ** *: ***** ***********:***.*****

MG04745.1 	IFSTIFREETDRVYGKVFIQEFVDARRR--AIQNAPQVLFRNDPPLELQGVPGVKDTGTG
NCU03050.1	IFSTIFREETDRVFGKVFIQEFVDARRR--AIQNAPQVLFRNDPPLELQNVPGVKNT---
FG07054.1 	IFSTVFREETDRVFGKVFIQEFVDARRR--AIQNAPQVLFRNDAPLELQGVPGIQNTGSG
AN0306.1  	IFSTVFREETDRIFGKVFLQEFVDARRRVATLQNAPQVLFRNDPPLELAGVPGLQDAGDG
          	****:*******::****:********* :::***********.**** .***::::. .

MG04745.1 	EIGYV-----------------TF~V1LFPRHLTPQRMPDVISHIQTFRDYFHYHIKASK
NCU03050.1	-----------------------A1V~LFPRHLTPQRMPDVISHIQTFRDYFHYHIKASK
FG07054.1 	DIGYVTFGKREKLLRLEAFTDRII~V~LFPRHLTPQRMTDVISHIQTFRDYFHYHIKASK
AN0306.1  	KISYIT-----------------F~V1LFPRHLTPQRRQENISHIQTFRDYFHYHIKASK
          	. .  :                   * **********  : *******************

MG04745.1 	0AYIHTRMRRRTADFLQV1LRRARPDSEEKERKTASGRTFKVQGN
NCU03050.1	0AYIHSRMRKRTADFLQV~LRRARPDTEEKEKKTASGRTFKAGA-
FG07054.1 	~AYIHSRMRKRTADFLQV1LRRARPENEEKERKTASGRTFKVQGN
AN0306.1  	0AYIHTRMRKRTADFLQV1LNRARPENEERERKTASGRTFRVQG-
          	 ****:***:******** *.****:.**:*:********:. .
```
